# Anti-GITR Antibody Treatment Increases TCR Repertoire Diversity of Regulatory but not Effector T Cells Engaged in the Immune Response Against B16 Melanoma

**DOI:** 10.1007/s00005-017-0479-1

**Published:** 2017-06-21

**Authors:** Bozena Scirka, Edyta Szurek, Maciej Pietrzak, Grzegorz Rempala, Pawel Kisielow, Leszek Ignatowicz, Arkadiusz Miazek

**Affiliations:** 1 Department of Tumor Immunology, Hirszfeld Institute of Immunology and Experimental Therapy, Polish Academy of Sciences, R. Weigla 12, 53-114 Wrocław, Poland; 2 Department of Biochemistry and Molecular Biology, Wroclaw University of Environmental and Life Sciences, Wrocław, Poland; 3 Center for Biotechnology and Genomic Medicine, Medical College of Georgia, Augusta, GA USA; 4 Mathematical Biosciences Institute, College of Public Health, Ohio State University, Columbus, OH USA

**Keywords:** GITR, DTA-1, TCR diversity, Immunotherapy, T regulatory cells, B16 melanoma

## Abstract

Crosslinking of glucocorticoid-induced TNF family-related receptor (GITR) with agonist antibodies restores cancer immunity by enhancing effector T cell (Teff) responses while interfering with intra-tumor regulatory T cell (Treg) stability and/or accumulation. However, how anti-GITR antibody infusion changes T cell receptor (TCR) repertoire of Teffs and Tregs engaged in anti-tumor immune response is unclear. Here, we used a transgenic mouse model (TCRmini) where T cells express naturally generated but limited TCR repertoire to trace the fate of individual T cells recognizing B16 melanoma in tumor-bearing mice, treated or non-treated with an anti-GITR monoclonal antibody DTA-1. Analysis of TCRs of CD4^+^ T cells from these mice revealed that the TCR repertoire of dominant tumor-reactive Teff clones remained rather similar in treated and non-treated mice. In contrast, both tumor-associated and peripheral TCR repertoire of Tregs, which were mostly distinct from that of Teffs, underwent DTA-1 mediated remodeling characterized by depletion of dominant clones and an emergence of more diverse, low-frequency clones bearing increased numbers of TCRs shared with Teffs. We conclude that the DTA-1 infusion eliminates activated Tregs engaged in the initial maintenance of tolerogenic niche for tumor growth, but over time, it favors tumor replenishment by Tregs expressing an array of TCRs able to compete with Teffs for recognition of the same tumor antigens which may prevent its complete eradication.

**Electronic supplementary material:**

The online version of this article (doi:10.1007/s00005-017-0479-1) contains supplementary material, which is available to authorized users.

## Introduction

In vivo stimulation of glucocorticoid-induced TNF family-related receptor (GITR) elicits an array of T cell responses ranging from proliferation to apoptosis (Ephrem et al. 2013) and results in a potent anti-tumor response to established tumors including murine B16 melanoma (Ramirez-Montagut et al. 2006; Turk et al. 2004). In general, GITR crosslinking with agonistic antibodies enhances effector responses of the conventional CD4^+^ and CD8^+^ T cells both in the setting of chronic viral infections or tumor cell growth (Clouthier et al. 2014; Dittmer et al. 2004; Ko et al. 2005). On the other hand, the effect of GITR signaling on the fate of regulatory T cells (Tregs) is less clear. Recent studies showed that both recombinant GITR ligand (GITR-L) or agonist anti-GITR antibody (DTA-1) treatment of tumor-bearing mice led to a loss of tumor-infiltrating Tregs due to either cell depletion, reduced intra-tumor infiltration, or intra-tumor down-regulation of the Foxp3 expression (Coe et al. 2010; Cohen et al. 2010; Hu et al. 2008). Yet, other reports underlined an important role of GITR signaling for Tregs proliferation, suppressor function (Liao et al. 2010, 2014), and the maintenance of effector T cell (Teff)/Treg ratio (van Olffen et al. 2009). The local cellular environment seems to influence the outcome of GITR signaling as its effect on tumor infiltrating versus tumor draining/peripheral lymph node Tregs may be different (Cohen et al. 2010). For example, in a number of solid tumor models, it was shown that the anti-GITR monoclonal antibody (mAb) targeted tumor infiltrating but not peripheral Tregs suggesting that local inflammation sensitizes these cells to DTA-1 mediated inactivation/depletion (Hindley et al. 2011; Sainz-Perez et al. 2012). Experiments involving a DTA-1 devoid of FcRγ binding capacities have shown that it is ineffective in controlling tumor growth (Bulliard et al. 2013), strongly suggesting that the antibody-mediated cytotoxicity of DTA-1 plays a predominant role in shifting an initial intra-tumor balance between the Tregs and Teffs in favor of the latter. Consistently, a true GITR signaling elicited by a recombinant pentameric GITR-L led only to a transient inhibition of MC38 tumor growth which was due to an enhancement of naïve Treg proliferation on the one hand, and on the other hand to a gradual accumulation of activated Tregs, which are insensitive to GITR mediated suppression (Kim et al. 2015). Therefore, dynamic changes of T cell proportions follow the DTA-1 infusion, but, due to complex effects elicited by this antibody, the directions of accompanying T cell receptor (TCR) repertoire changes and their significance for tumor eradication are largely unknown. For example, it has been proposed that the DTA-1 based immunotherapy enhances the polyclonality of tumor antigen-specific responses (Ramirez-Montagut et al. 2006; Schaer et al. 2012), but several questions remain. First, whether both tumor-specific Teff and Treg clones increase in size and complexity or only Teffs or Tregs? Second, does the anti-GITR therapy influence conversion of Teffs into Tregs within tumor environment (Hindley et al. 2011; Kuczma et al. 2010)? Third, in the light of a prevailing view that Teffs and Tregs recognize distinct tumor antigens (Bonertz et al. 2009; Hindley et al. 2011), it is interesting if tumor-associated neo-antigen(s) promote expansion of Teffs or Tregs or both and whether these subsets express a similar or different TCRs specific for this neo-antigen(s)? To address these questions, we took advantage of TCRmini^Foxp3GFP^ (TCRmini) mice in which GFP marks only Tregs and their limited TCR diversity allows for a comprehensive assessment of expansion and recruitment of tumor-specific T cells infiltrating tumor and tumor draining lymph nodes at the level of individual clones (Kuczma et al. 2009, 2010; Pacholczyk et al. 2006). We found that the DTA-1 treatment only minimally affected the TCR diversity and overall frequency of dominant tumor--reactive CD4^+^Foxp3^−^Teff clones (Teffs). By contrast, the frequency of tumor-infiltrating CD4^+^Foxp3^+^Tregs with the same TCR specificities as Teffs dropped by over 50% upon the DTA-1 treatment, but the unexpected diversity of their TCRs increased at the levels of amino acid and nucleotide sequences. These observations suggest that therapeutic effect of anti-GITR-based immunotherapy depends on ablation of an initial pool of tumor-infiltrating Tregs which can be later replaced by a more diverse but minor population of Tregs bearing TCRs able to compete with Teffs for recognition of tumor-associated antigens and thus be responsible for a failure to eradicate the tumor completely.

## Results

### Treatment with DTA-1 Results in Regression of B16 Melanoma in TCRmini Mice

**Fig. 1:**
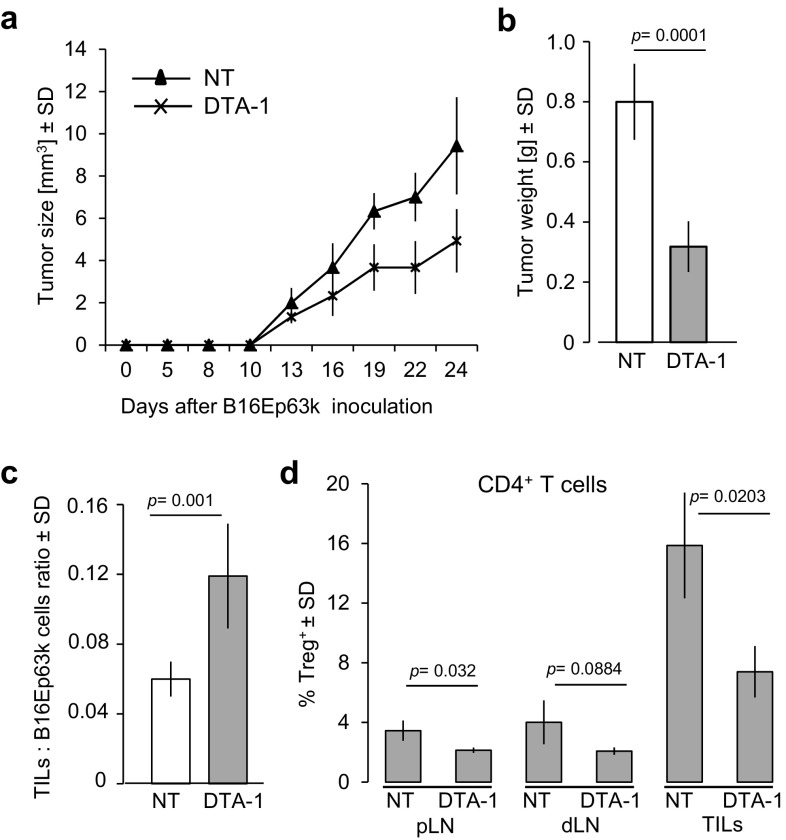
Effects of DTA-1 treatment of tumor-bearing TCRmini mice. Eight days after subcutaneous injection of the B16-Ep63K melanoma, mice (*n* = 12) were intravenously infused with a single dose (1 mg) of DTA-1. **a** Kinetics of tumor growth in DTA-1 treated and untreated (NT)—i.e., infused with normal rat Ig—mice. **b** Tumor weight at day 24. **c** The ratio of tumor-infiltrating T lymphocytes (TILs) to B16-Ep63K cells and **d** percentage of Tregs in total CD4^+^T cells in peripheral lymph nodes (pLN), draining lymph nodes (dLN), and TILs of untreated or DTA-1 treated mice

To analyze the influence of DTA-1 treatment on tumor growth and accompanying changes in composition of functionally different subsets in populations of CD4^+^ T cells infiltrating the tumor (TILs) and different lymph nodes, we used TCRmini mice (Kuczma et al. 2009; Pacholczyk et al. 2006) bearing B16 melanoma genetically engineered to express the peptide neo-antigen Ep63K (B16-Ep63K). The TCR repertoire of TCRmini mice contains multiple TCRs specific for Ep63K, because the TCR that served as a template to generate TCRmini constructs was cloned from CD4^+^ T cells specific for this antigen (Kuczma et al. 2010). To evaluate the therapeutic effect of DTA-1 on an established tumor, we administrated the antibody on day 8 after injection of the tumor. Pilot experiments showed that this protocol led to a strong inhibition of the B16-Ep63K tumor growth but unlike repeated injections on day 4 and 8 (data not shown), it did not cure the mice. As shown in Fig. 1a and b, the tumor diameter and weight on day 24 post-injection were reduced by 50–60% (*p* = 0.0001) in DTA-1 treated mice in comparison to control mice receiving normal rat immunoglobulin. The inhibition of tumor growth was accompanied by increased ratio of TILs to B16-Ep63K tumor cells (Fig. 1c), but the proportion of Tregs among TILs and lymph node T cells decreased, while the proportion of CD4^+^Teffs increased (Fig. 1d). Notably, most tumor-infiltrating Tregs and CD4^+^Teffs displayed activated (CD62L^low^) phenotype irrespectively of the DTA-1 treatment (Supplementary Fig. 1a) and elevated GITR expression (Supplementary Fig. 1b), suggesting that TCRs of these cells have been triggered.

### The Influence of the Tumor on TCR Repertoire of CD4^+^Teffs and Tregs

**Fig. 2:**
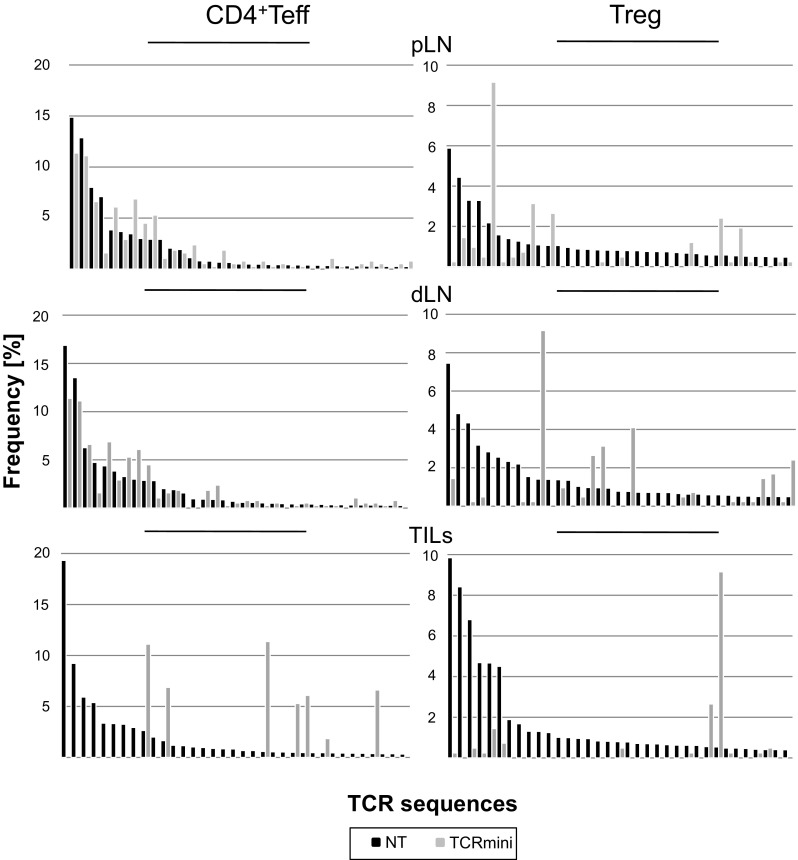
Comparison of dominant TCRs expressed by effector and regulatory CD4^+^ T cells in healthy and tumor-bearing TCRmini mice. The influence of the tumor on TCR repertoire of CD4^+^Teffs and Tregs. The frequencies of 35 most dominant TCRs from CD4^+^Teffs and Tregs from indicated sites of B16-Ep tumor-bearing (*black bars*) mice in comparison to TCRs present among circulating lymphocytes of healthy non-tumor-bearing control TCRmini mice (*gray bars*)

To assess the degree of TCR repertoire skewing toward tumor antigens in various organs of tumor-bearing mice and to evaluate the contribution of public clones to anti-tumor response, we compared the frequency of dominant TCRs of CD4^+^ T cells from these mice with their frequency in normal healthy TCRmini mice. For this purpose, we plotted the frequencies of 35 most frequent TCRs expressed by CD4^+^Teffs and Tregs isolated from lymph nodes or infiltrating the B16-Ep63K tumor, against their frequencies in the total repertoire of healthy, non-tumor-bearing TCRmini mice (untreated). The results shown in Fig. 2 indicate that in peripheral (pLN) and tumor draining (dLN) lymph nodes, most TCRs found on dominant CD4^+^Teff clones in tumor-bearing mice were also found with similar frequency in the repertoire of non-tumor-bearing, healthy TCRmini mice. On the other hand, only about 20% of TCRs found on dominant 35 CD4^+^Teff clones in tumor infiltrates were also found among circulating lymphocytes of control, non-tumor-bearing mice albeit at different frequency. In contrast to CD4^+^Teffs, the proportion and the frequency of Treg clones sharing the same TCR were very different not only among tumor infiltrates, where the differences were most pronounced but also in dLN and pLN. Thus, we concluded that in the lymph nodes, much stronger remodeling of TCR repertoire was observed in the case of Tregs than CD4^+^Teffs, whereas the repertoires of CD4^+^Teffs and Tregs among TILs were similarly affected, mostly due to clonal expansion of low frequency or new clones.

### Effect of DTA-1 Treatment of Tumor-Bearing Mice on the TCR Repertoire of CD4^+^Teffs and Tregs

**Fig. 3:**
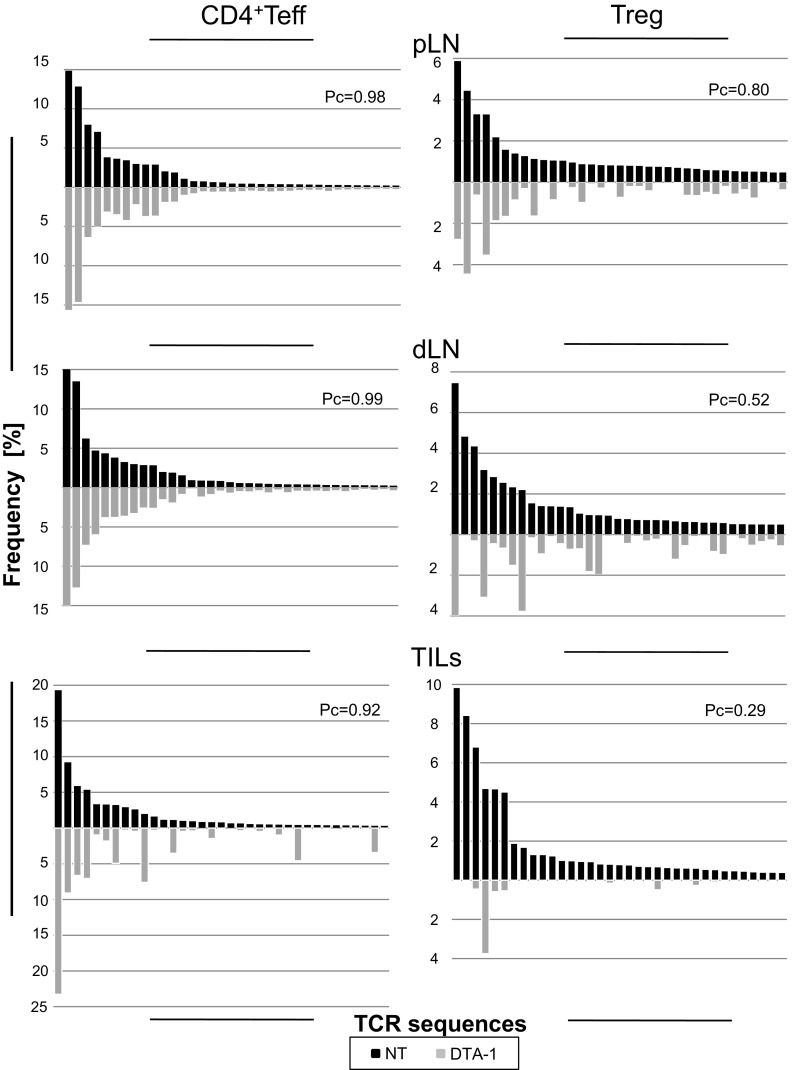
Influence of DTA-1 treatment on TCR repertoires of CD4^+^Teffs and Tregs in indicated anatomical sites of tumor-bearing TCRmini mice. *Black bars* and *gray bars* correspond to non-treated and DTA-1 treated mice, respectively. Pearson’s coefficient (Pc) indices are given above each graph

It has been shown that infusion of DTA-1 into tumor-bearing mice changes the composition of the Treg population by depleting activated Tregs (GITR^hi^) and by promoting the expansion of naïve Tregs (Kim et al. 2015). To analyze how DTA-1 treatment influences the clonal diversity of CD4^+^Teffs and Tregs in tumor-bearing TCRmini mice, we first compared the frequencies of 35 most frequent clones in pLN, dLN, and tumor from untreated and DTA-1 treated mice. Figure 3 shows that, in lymph nodes, DTA-1 treatment mostly changed frequencies of Treg clones, whereas the frequencies of dominant CD4^+^Teff clones remained almost unchanged. Significantly, DTA-1 treatment changed the frequency of many dominant Treg and CD4^+^Teff clones in tumor infiltrates, Tregs being more strongly affected than CD4^+^Teffs. Most (80%) of analyzed Treg clones found in tumors in untreated mice were undetectable among Tregs retrieved from DTA-1 treated tumors, and the remaining clones had low frequency. Interestingly, frequencies of 7 (20%) of CD4^+^Teff clones in untreated mice increased significantly following DTA-1 treatment, whereas no such clones were detected in Treg population. For CD4^+^Teffs from all anatomical sites, the Pearson’s coefficient (Pc) values reflecting the degree of similarity between two sets of variables were high with an exception of TILs in which low abundant clones were affected by the DTA-1. In contrast, the Pc values for Tregs were much lower indicating that DTA-1 treatment significantly altered clonotype frequencies within the latter but not within the former population.

**Fig. 4:**
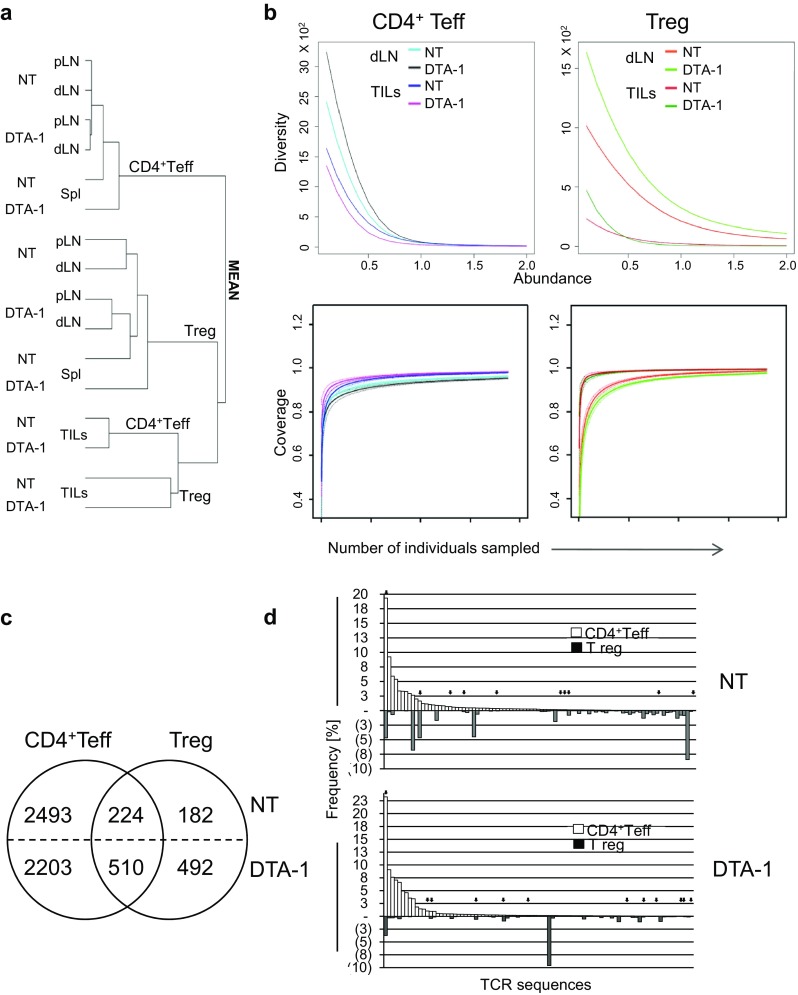
Comparison of similarity between **a** TCR repertoires of Teff and Treg clones from lymph nodes (dLN and pLN), spleen (Spl), and tumor infiltrates (TILs) from untreated (NT) and DTA-1 treated TCRmini mice. **b** Diversity and coverage of TCRs from CD4^+^Teff and Treg clones. The hierarchical charts depict similarity indices for TCR repertoires based on high-throughput CDR3 sequencing. **c** *Circle charts* show numbers of different TCRs shared by CD4^+^Teffs and Tregs or expressed only on each of these subsets separately among 2 × 10^4^ TILs from NT or DTA-1 treated tumor-bearing TCRmini mice. **d** Frequency of 100 dominant TCRs of CD4^+^Teff TILs (*open bars*) and corresponding Treg TIL clones (*filled bars*) from NT (*upper panel*) and DTA-1 treated (*lower panel*) tumor-bearing TCRmini mice. Arrows mark TCRs also found on T cell hybridomas derived from the same pool of cells, which responded ex vivo to B16 tumor antigens

To analyze the complexity of TCR repertoire of lymph node and tumor-infiltrating CD4^+^Teffs and Tregs and to determine a proportion of common TCRs in treated and non-treated mice, we compared TCRs from these functionally different T cells (Fig. 4). Hierarchical clusters, depicting similarity of TCR repertoires, placed CD4^+^Teff and Treg TILs in close proximity, and separate from CD4^+^Teffs and Tregs found in various lymphoid organs including dLN, which were placed on separate branches (Fig. 4a), suggesting that the majority of CD4^+^TILs expressed TCRs that are relatively infrequent. The assessment of global TCR complexity revealed that DTA-1 treatment in case of dLN increased overall diversity of both Tregs and CD4^+^Teffs, but in the tumor, only diversity of Tregs—mainly those of low abundance—increased, while the diversity of Teffs decreased (Fig. 4b). Possibly, this could be explained by an ablation of dominant Treg clones in tumor milieu and their replacement by rare thymus-derived Treg clones or conversion of certain CD4^+^Teffs. Indeed, comparison of the number of different clones among Teffs and Tregs identified within the same (2 × 10^4^) number of TILs from untreated and treated mice also confirmed that DTA-1 treatment increased the diversity of Treg TILs resulting in a higher proportion of common TCRs (224 vs. 510) (Fig. 4c). Since the similarity indices between the TILs Treg and dLN Treg from treated mice were low, this result also (in reference to above statement about the diversity) implied that DTA-1 eliminated locally intra-tumor, dominant Treg clones rather than enhanced the recruitment of new Tregs from dLN. CD4^+^Teff and Treg clones expressing identical TCRs likely recognize the same antigens, but their activation may have a different effect on the growth of the tumor. To get insight into the direction of changes in the frequency of CD4^+^Teff and Treg TILs expressing common TCRs, which may accompany tumor regression, we analyzed the abundance of such clones in untreated and DTA-1 treated mice. We plotted 100 TCRs that were dominant on CD4^+^Teff TILs and shared with Treg TILs separately from untreated or DTA-1 treated mice and assessed for frequencies of the corresponding TCRs. The results are shown in Fig. 4d demonstrating that following DTA-1 treatment, the frequencies of Treg clones, and particularly those that corresponded to the most frequent CD4^+^Teff clones, dropped down. Moreover, the TCRs of most frequent CD4^+^Teff clones, whose frequencies were comparable or higher among Tregs, were absent after the DTA-1 treatment. This result directly implied that the DTA-1 exerted opposite effects on populations of effector and Tregs sharing the same TCRs of unknown or tumor-reactive specificity (Fig. 4c, arrows).

### Identification of B16Ep63K Tumor-Reactive TCRs

CD4^+^ T cells proliferating in response to inoculated tumor may include antigen-specific as well as “bystander-activated” clones. To identify tumor-specific clones that recognize Ep63K neo-antigen or native B16 antigens, we immortalized CD4^+^Teffs and Tregs sorted from tumor mass or tumor draining lymph nodes of both untreated and DTA-1 treated TCRmini mice. In sum, sixteen B16 melanoma-reactive T cell hybridomas were generated from CD4^+^Teffs and Tregs, sorted from tumor mass or tumor draining lymph nodes of both untreated and DTA-1 treated TCRmini mice. Supplementary Figure 2 shows that two of these hybridomas were exclusively specific for Ep63K neo-antigen, whereas the rest responded to unknown tumor antigens shared by B16 and B16Ep63K melanomas. To determine how often these TCRs are found in the normal repertoire of healthy TCRmini mice, we cross-referenced their sequences to the database of CD4^+^TCRs from this model (Supplementary Table 1). We found that all TCR sequences identified as tumor reactive were present on both CD4^+^Teffs and Tregs, although with various frequencies. This finding suggested that Tregs and Teffs expressing TCR of the same specificity could at least in part arise by conversion from CD4^+^Foxp3^−^ T cells or that Tregs were derived from separate precursors or that many tumor-reactive clones pre-exist in normal repertoire, often at relatively high frequency, and that their TCRs do not exclusively drive these cells differentiation to only effector or regulatory lineages.

**Fig. 5:**
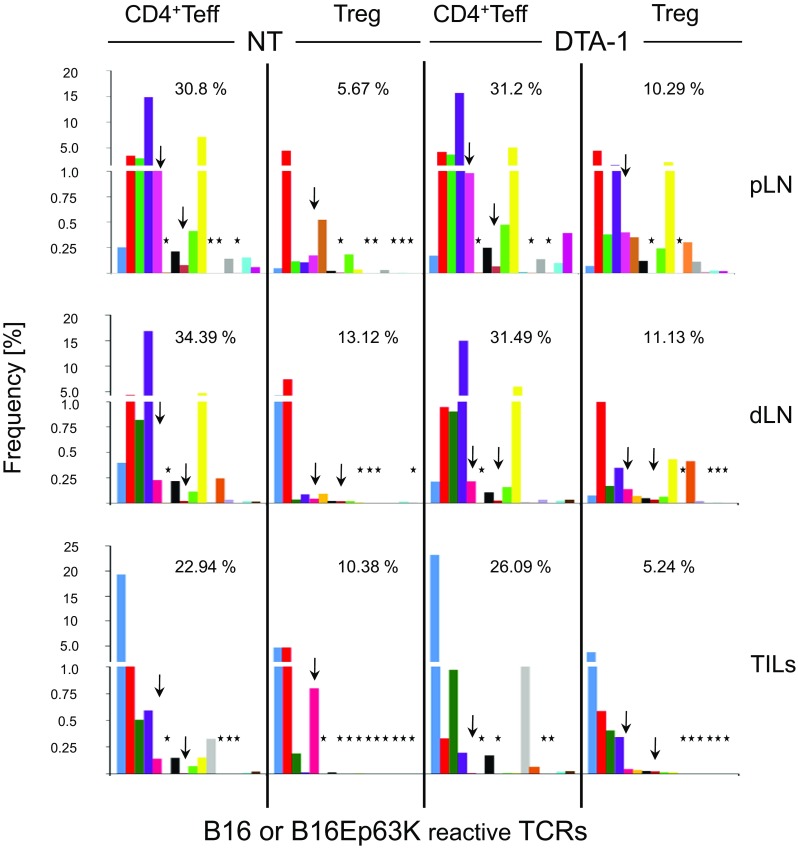
Frequencies of 16 B16 reactive TCRs: two of them specific for Ep63K neo-antigen (*arrows*) among pLN, dLN, and TIL CD4^+^ T cells. *Bars* represent given TCR (marked by *different colors*) expressed on CD4^+^Teff or Treg clones from tumor-bearing untreated (NT) or DTA-1 treated mice. The absence of given clone is marked by an *asterisk*

Interestingly, added frequencies of individual tumor-reactive TCRs in pLN, dLN, and TILs amounted to 22–34% of all CD4^+^Teff clones, but only to 5–13% of Treg clones in respective anatomical locations (Fig. 5). DTA-1 treatment of tumor-bearing mice had a different impact on CD4^+^Teffs and Tregs. In the case of Teffs, the added frequency in different sites of untreated and treated mice remained similar. In contrast, changes in the Treg frequencies depended on their anatomical localization. In pLN, DTA-1 infusion increased frequencies of tumor-reactive Treg clones in pLN but reduced in the tumor. This observation applied to T cell clones specific for both autonomous or Ep63K neo-antigens (Fig. 5) and was consistent with the observed biological effect of Tregs depletion on tumor growth following the DTA-1 treatment (see Fig. 1d).

Next, we assessed that the diversity of TCR repertoires of tumor-specific CD4^+^Teffs and Tregs in pLN, dLN, and tumor in treated and untreated mice was assessed by counting numbers of tumor-reactive CD4^+^Teffs and Tregs clones. First, this analysis revealed that an overall diversity of TCRs in pLN and dLN was higher than in tumor although lymph nodes contain more T cells than the tumor. Second, following DTA-1 treatment, the most prominent changes in the diversity were observed in the case of pLN and TIL Tregs for which DTA-1 treatment increased the number of individual clones 9–14 and 6–10, respectively. The increase in TIL Treg diversity concerned particularly clones of lower frequency (Fig. 5, lowest right panel).

### Analysis of Diversity of Teff and Treg Clones Sharing the Same Tumor-Reactive TCR

**Fig. 6:**
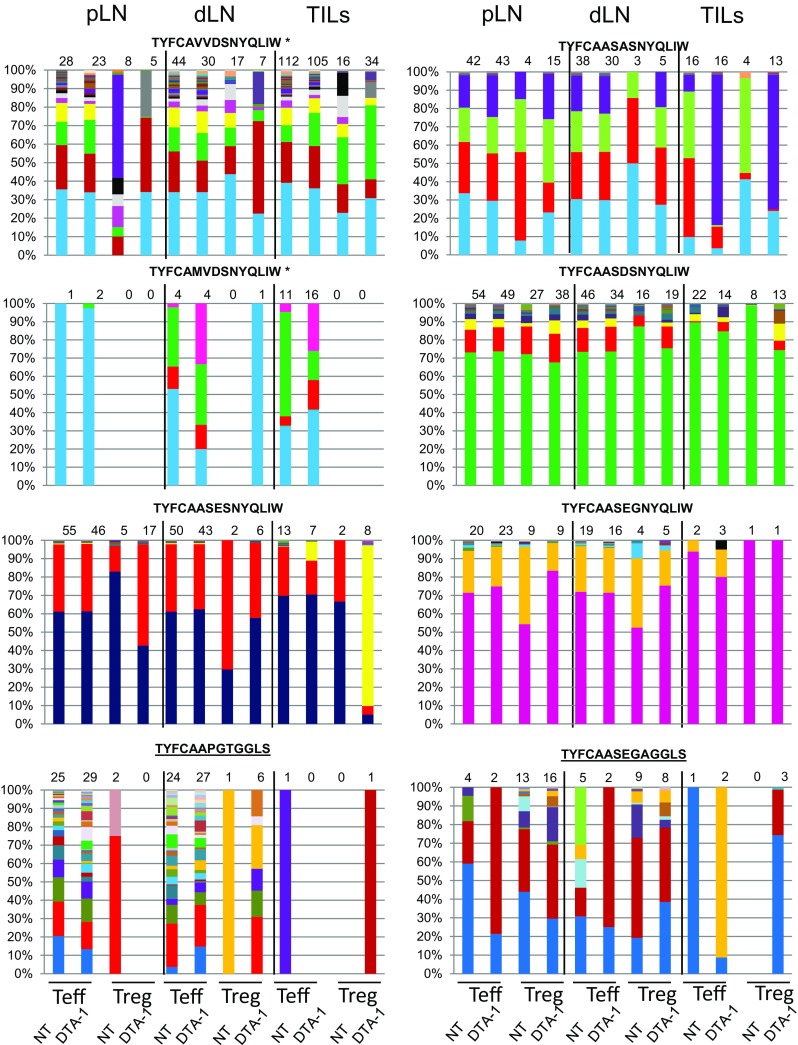
Distribution and frequency of single nucleotide sequences encoding six B16 tumor-reactive (three *upper panels*) and two Ep63K-specific (*lowest panel*) TCRs. Amino acid sequences of TCRα CDR3 region are given above each graph. The TCRs were chosen according to their highest frequency within tumor-infiltrating (TILs) CD4^+^Teffs population. *Color-coded bars* represent the distribution of single nucleotide sequences encoding given TCR within T cells from peripheral lymph nodes (pLN) draining lymph nodes (dLN) or TILs of untreated (NT) or DTA-1 treated mice. *Numbers* above each graph indicate the total number of nucleotide sequences encoding a given TCR. *Asterisk* indicates TCR sequences whose diversity in the tumor was higher than in pLN

To get insight into the mechanism underlying changes in the diversity of pLN, dLN, and TIL Teffs and Tregs sharing the same TCR identified by tumor-reactive hybridomas, nucleotide sequences encoding six most frequent non-Ep63K specific and two Ep63K specific TCRs (Fig. 6, bottom panels) were analyzed. We found that a number of different nucleotide sequences encoding a given TCR varied 1–112. Most of the high-frequency TCR clonotypes were found on both Teffs and Tregs suggesting that Teff to Treg conversion can, at least in part, explain the increase of Treg diversity. On the other hand, we detected a number of infrequent clonotypes, unique to Tregs and Teffs (see Supplementary Table 2). Such nucleotide sequences were enriched in pLNs and, in particular, were found among TCRs whose frequency at the aa level dropped on the Teffs upon the DTA-1 treatment (Supplementary Table 1). These findings suggest that clonal expansion of Teffs may occur in parallel to conversion to Tregs, whereas DTA-1-mediated depletion or inhibition of Teffs growth may promote recruitment of more diverse pool of thymus-derived Treg clones.

## Materials and Methods

### Mice

The TCRmini and C57BL/6 (B6)Foxp3^GFP^ mice were used in experiments TCRmini mice that harbor a mini-repertoire of TCR α chains encoded by Vα2.9 and Jα26 (or Jα2) associated with one rearranged TCR Vβ14 chain (Kuczma et al. 2009; Pacholczyk et al. 2006). The mice were screened for the co-expression of Foxp3^GFP^ reporter and TCRmini Vα2 Vβ14^+^ dimer or TCRβ Vβ14^+^ chain. All TCRmini mice were crossed with mice deficient in endogenous TCRα loci and were heterozygous for TCRα Vα2Jα26Jα2 minilocus to ensure expression of a single TCRα chain per T cell. The diversity of the TCR repertoire depends on the CDR3 region of the TCRα chain. All animals were housed in an animal facility in accordance with the institutional regulations. All animals were 6–12 weeks old in our experiments. All procedures using mice were approved by the First Local Ethical Commission in Wroclaw, under permission number 06/2009.

### Tumors

The mouse melanoma cell line B16 was obtained from Dr. Kraj (GHSU, USA). For experiments, a B16 melanoma was used which expressed Ep63K peptide on the cell surface, as part of the influenza virus nucleoprotein. To prepare the cells for inoculation, B16 melanoma cells expressing Ep63K-GFP were sorted and expanded in vitro for 2–3 days before injection. Tumor cells (5 × 10^4^) were injected s.c. inside of one thigh of 6–12-week-old TCRmini and B6Foxp3^GFP^ mice. Mice were treated DTA-1 mAb after tumor challenge (8th day) and were analyzed after 24 days. Lymph nodes, tumors, and spleens from mice were pooled for TCR repertoire analyses.

### Monoclonal Antibody

DTA-1 is a rat IgG2b mAb specific for mouse GITR (a kind gift of Dr. Shimon Sakagouchi) (Ko et al. 2005). This mAb was partially purified from hybridoma culture supernatants by precipitation in 45% ammonium sulfate, dialyzed against PBS, and further purified using protein G column. DTA-1 treatment was conducted by intraperitoneal injection (1 mg/mouse) 8 days after tumor inoculation.

### T Lymphocyte Purification

Single-cell suspensions of peripheral T cells were prepared from control lymph nodes (pLN), draining lymph nodes (dLN), and spleens originating in tumor-bearing mice (untreated and DTA-1 treated). These organs were dissected, ground through 70 μm cell strainer, and washed twice in PBS + 2% fetal bovine serum (FACS solution). Then, cells were used for flow cytometry or sorting. TILs were prepared from tumor lesions by scrubbing tumor tissue into PBS with 0.1 M EDTA. B16 cell suspension was stained with mAbs for flow cytometry analysis and sorting.

### Multiparameter Flow Cytometry

T lymphocytes were stained with antibodies against CD4, CD8α, Vα2, Vβ14, CD62L, and GITR (BD Biosciences, USA or eBioscience, Austria), and analyzed. Cell-surface staining with mAbs was done by standard procedure (Laszkiewicz et al. 2011). Purification of cell subsets by flow cytometry was done using BD Aria cell sorter (Beckton Dickinson, USA) according to the manufacturer’s instructions. Single cells were sorted from indicated organs on populations: CD4^+^Foxp3^GFP+^; CD4^+^Foxp3^GFP−^ with purity >98%. Flow cytometry was performed on BD Calibur or BD LSRFortessa apparatus according to standard protocols. FACS data were analyzed with BD CellQuest software.

### High-Throughput CDR3 Sequencing

Analysis of the TCRmini Va2Ja26Ja2 locus in tumor-bearing mice (untreated and DTA-1 treated) was performed from flow cytometer-purified CD4^+^Foxp3^GFP−^, CD4^+^Foxp3^GFP+^, CD8 T cells (>98%). RNA was isolated (RNeasy Mini/Micro Kit, Qiagen, Germany) and converted to cDNA (SuperScript III, Invitrogen, USA) with Cα-specific primer (5′-TCGGCACATTGATTTGGGAGTC-3′). TCRα CDR3 regions were amplified using primers with incorporated tags for IonTorrent sequencer (Vα2IT: 5′-CCATCTCATCCCTGCGTGTCTCCGACTCAGTCTCAGCCTGGAGACTCAGC-3′ and CαIT: 5′-CCTCTCTATGGGCAGTCGGTGATTGGTACACAGCAGGTTCTGGGT-3′). The PCR product was sequenced by EdgeBio (Gaithersburg, MD, USA). CDR3 regions sequenced on the same chip and derived from different subsets were discriminated based on barcodes, which were validated for optimal performance with the Ion Torrent PGM.

### Hybridoma Assays

T lymphocytes (CD4^+^Foxp3^GFP−^ and CD4^+^Foxp3^GFP+^) from dLN and tumor were expanded in vitro for 7 days and fused with BW thymoma stably transfected with the NFAT^GFP^. After 10 days, monoclonal hybridomas were propagated and measured their response using flow cytometry and ELISA assays. In brief, 10^5^ hybridoma cells were incubated with B16 or B16Ep63K cell suspensions. After 24 h, the GFP fluorescence was monitored by BD FACS Calibur and the amount of secreted IL-2 was measured in triplicates using Novex mouse IL-2 ELISA kit (Life Technologies, Carlsbad, USA). The TCRα CDR3 regions from responding hybridomas were amplified, sequenced, and cross-referenced to our database of TCRα CDR3 collected from subpopulations of CD4^+^Foxp3^GFP−^ and CD4^+^Foxp3^GFP+^ cells in tumor-bearing mice (untreated or DTA-1 treated) and control unmanipulated TCRmini mice.

### Statistics

The bar charts indicate the mean ± SD. The graphed data are representative of at least three biological replicates. Student’s *t* test was used to analyze the FACS data from FACS data. *P* ≤ 0.05 was considered to indicate a statistically significant difference. The asterisk denotes statistically significant differences between the indicated samples. Pearson’s coefficient was calculated for sets of TCR frequencies using Microsoft Excel software. Diversity of the TCR repertoires was calculated using the method described by Chao et al. (2006).

## Discussion

In this study, we used the TCRmini mouse model for assessing changes in CD4^+^Teff and Treg TCR repertoires following experimental anti-tumor immunotherapy with the anti-GITR antibody. By applying high-throughput DNA sequencing, we were able to identify TCRs of tumor-reactive CD4^+^Teffs and Tregs. Unexpectedly, all of 16 identified TCRs, including two Ep63K specific ones, were present at different frequencies in both Teff and Treg populations (Supplementary Table 1). Because of the lack of definitive markers of conversion, it was impossible to conclude if these common TCRs are a result of Teff to peripheral Treg conversion or selection of thymus-derived Treg clones from separate precursors. Analysis of single clonotype expansions of these common TCRs (Fig. 6; Supplementary Table 2) indicated that the majority of them were present within the tumor, supporting conversion, although a few, especially in peripheral lymph nodes, were found only among Tregs suggesting that both phenomena may account for the observed overlap in the TCR usage.

The use of a therapeutic antibody of certain isotypes is linked with direct, complement mediated, and/or cell-mediated cytotoxicity (Brunn et al. 2016; Murphy et al. 2014). For the DTA-1 mAb, it is known that binding to its target elicits cytolytic effects (Coe et al. 2010) that are proportional to the density of GITR on the cell surface. The highest expression level of GITR is reportedly found on the surface of activated Tregs (Supplementary Fig. 1b) including activated memory CD44^hi^ Tregs that constitute the first population to sense tumor presence by proliferating and increasing activation markers (Darrasse-Jeze et al. 2009). Accordingly, we observed that DTA-1 treatment led to a partial depletion of Tregs in all lymphoid organs including pLN (Fig. 1d) where coincidently it induced a significant increase in the frequency and diversity of tumor-reactive Tregs (Fig. 5, upper right panel). Opposite effect could be seen in the tumor where while increasing the diversity, DTA-1 strongly decreased Treg’s frequency (Fig. 5, lower right panel). The latter observation may reflect the differential influence of inflammatory environment/cellular context characterizing tumor tissue on the Treg sensitivity to DTA-1 depletion (Ephrem et al. 2013).

DTA-1 treatment did not further increase polyclonality of Teff response neither when assessing global diversity (Fig. 4a) nor when looking at tumor-reactive clones (Fig. 5). We observed that once set, the repertoire of Teffs involved in anti-tumor response remained roughly constant with only a few low abundant clones whose frequency changed. In contrast, TCR diversity of Tregs increased (Figs. 4a, 5). This observation is consistent with a reported effect of GITR engagement promoting proliferation of naïve but not activated Treg clones (Kim et al. 2015).

Our data also showed that tumor-associated neo-antigen Ep63K can be recognized by TCRs shared by Teffs and Tregs. Therefore, both T cell populations can compete for the recognition of the same tumor antigen.

Data presented in this report point to a previously unappreciated adaptation mechanism that unfolds upon depletion of a dominant Tregs involved in controlling effector anti-tumor response. On the basis of our results, we would like to suggest that antibody-mediated cytotoxicity and triggering of GITR signaling result in profound remodeling of Treg but not Teff intra-tumor and peripheral cell compartments. Because the single-dose infusion of DTA-1 slowed down tumor growth but did not eradicate it completely, we speculate that the failure to reject the tumor, in this case, may be due to the increased TCR diversity of the second wave of newly emerged Tregs which could better compete with Teffs for recognition of the same tumor antigens.

## Electronic supplementary material

Below is the link to the electronic supplementary material.

Supplementary material 1 (PPT 231 kb)

Supplementary material 2 (PPT 131 kb)

Supplementary material 3 (PPT 135 kb)

Supplementary material 4 (PPT 1222 kb)
