# Supplementary material for: Anti-GITR Antibody Treatment Increases TCR Repertoire Diversity of Regulatory but not Effector T Cells Engaged in the Immune Response Against B16 Melanoma
Source: Arch Immunol Ther Exp (Warsz). 2017 Jun 21;65(6):553–64. doi: 10.1007/s00005-017-0479-1 (PMC5688217; doi:10.1007/s00005-017-0479-1)
Supplement: Supplementary file 1 — Supplementary material 1 (PPT 231 kb) [file 5_2017_479_MOESM1_ESM.ppt]

## Slide 1
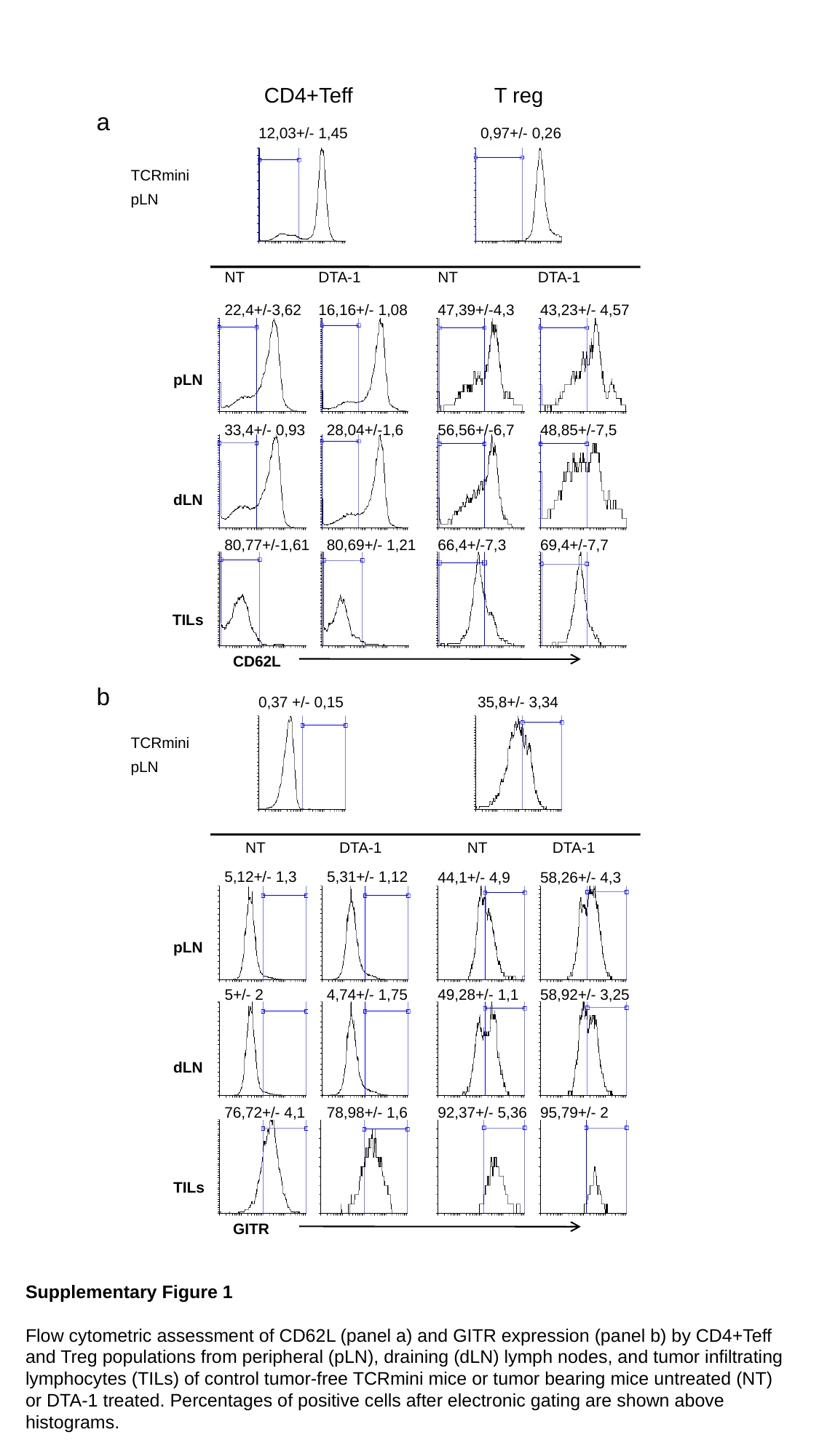

T reg
NT
DTA-1
NT
DTA-1
CD4+Teff
12,03+/- 1,45
0,97+/- 0,26
TCRmini
pLN
22,4+/-3,62
16,16+/- 1,08
47,39+/-4,3
43,23+/- 4,57
pLN
33,4+/- 0,93
28,04+/-1,6
56,56+/-6,7
48,85+/-7,5
dLN
80,77+/-1,61
80,69+/- 1,21
66,4+/-7,3
69,4+/-7,7
TILs
CD62L
a
b
0,37 +/- 0,15
35,8+/- 3,34
TCRmini
NT
DTA-1
NT
DTA-1
5,12+/- 1,3
5,31+/- 1,12
44,1+/- 4,9
58,26+/- 4,3
pLN
5+/- 2
4,74+/- 1,75
49,28+/- 1,1
58,92+/- 3,25
dLN
76,72+/- 4,1
78,98+/- 1,6
92,37+/- 5,36
95,79+/- 2
TILs
GITR
pLN
Supplementary Figure 1
Flow cytometric assessment of CD62L (panel a) and GITR expression (panel b) by CD4+Teff and Treg populations from peripheral (pLN), draining (dLN) lymph nodes, and tumor infiltrating
lymphocytes (TILs) of control tumor-free TCRmini mice or tumor bearing mice untreated (NT) or DTA-1 treated. Percentages of positive cells after electronic gating are shown above histograms.
