# Supplementary material for: Anti-GITR Antibody Treatment Increases TCR Repertoire Diversity of Regulatory but not Effector T Cells Engaged in the Immune Response Against B16 Melanoma
Source: Arch Immunol Ther Exp (Warsz). 2017 Jun 21;65(6):553–64. doi: 10.1007/s00005-017-0479-1 (PMC5688217; doi:10.1007/s00005-017-0479-1)
Supplement: Supplementary file 2 — Supplementary material 2 (PPT 131 kb) [file 5_2017_479_MOESM2_ESM.ppt]

## Slide 1
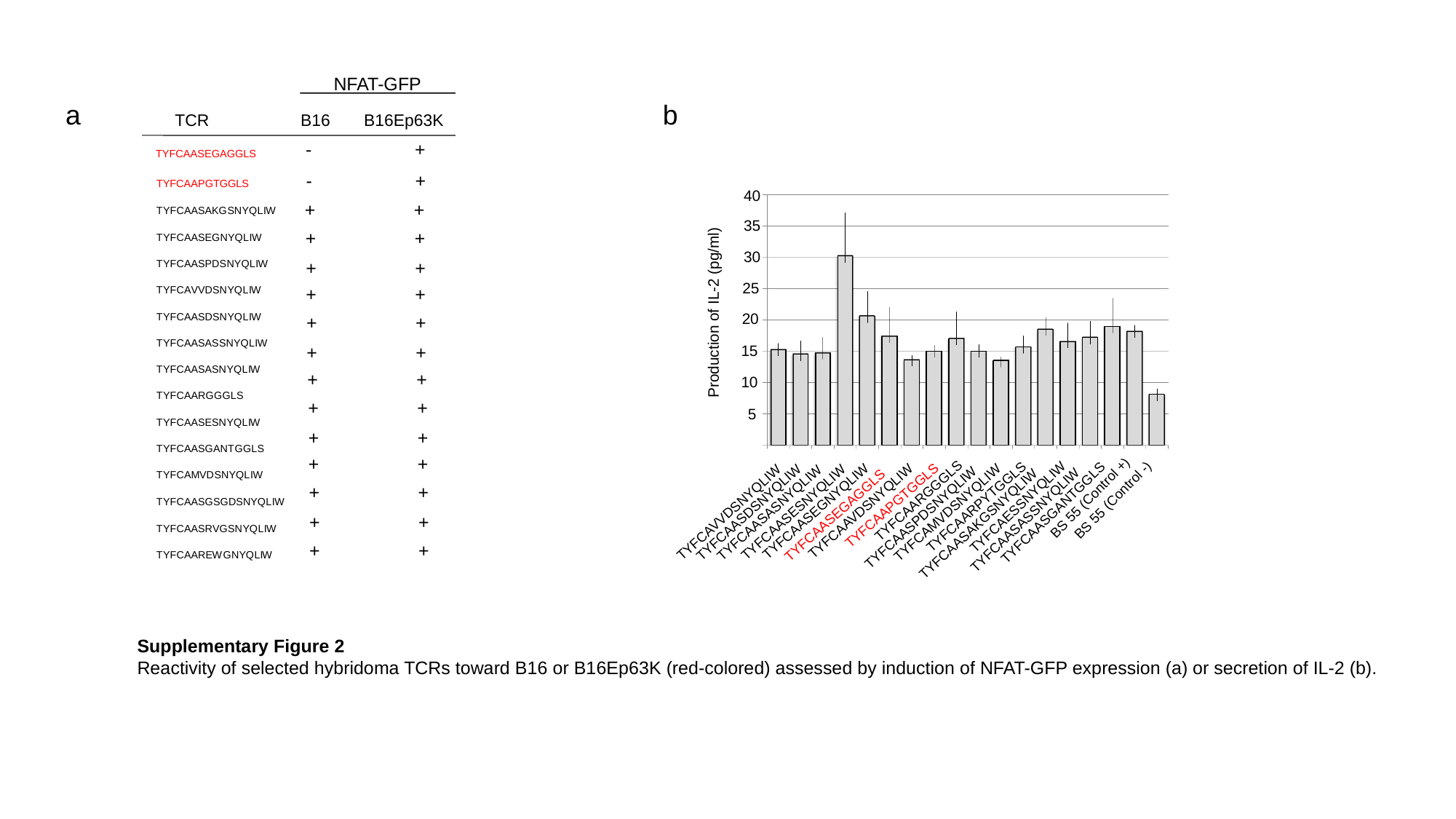

Production of IL-2 (pg/ml)
TYFCAARGGGLS
TYFCAESSNYQLIW
TYFCAAPGTGGLS
TYFCAASGANTGGLS
TYFCAAVDSNYQLIW
TYFCAASEGNYQLIW
TYFCAASESNYQLIW
TYFCAASDSNYQLIW
TYFCAASASSNYQLIW
TYFCAASASNYQLIW
TYFCAASEGAGGLS
TYFCAMVDSNYQLIW
BS 55 (Control +)
TYFCAVVDSNYQLIW
TYFCAASAKGSNYQLIW
TYFCAASPDSNYQLIW
TYFCAARPYTGGLS
BS 55 (Control -)
NFAT-GFP
a
b
TCR
B16
B16Ep63K
-	+
TYFCAASEGAGGLS
-	+
TYFCAAPGTGGLS
40
+	+
35
+	+
30
+	+
25
+	+
20
+	+
+	+
15
+	+
10
+	+
5
+	+
+	+
+	+
+	+
+	+
Supplementary Figure 2
Reactivity of selected hybridoma TCRs toward B16 or B16Ep63K (red-colored) assessed by induction of NFAT-GFP expression (a) or secretion of IL-2 (b).
