# Supplementary material for: Anti-GITR Antibody Treatment Increases TCR Repertoire Diversity of Regulatory but not Effector T Cells Engaged in the Immune Response Against B16 Melanoma
Source: Arch Immunol Ther Exp (Warsz). 2017 Jun 21;65(6):553–64. doi: 10.1007/s00005-017-0479-1 (PMC5688217; doi:10.1007/s00005-017-0479-1)
Supplement: Supplementary file 3 — Supplementary material 3 (PPT 135 kb) [file 5_2017_479_MOESM3_ESM.ppt]

## Slide 1
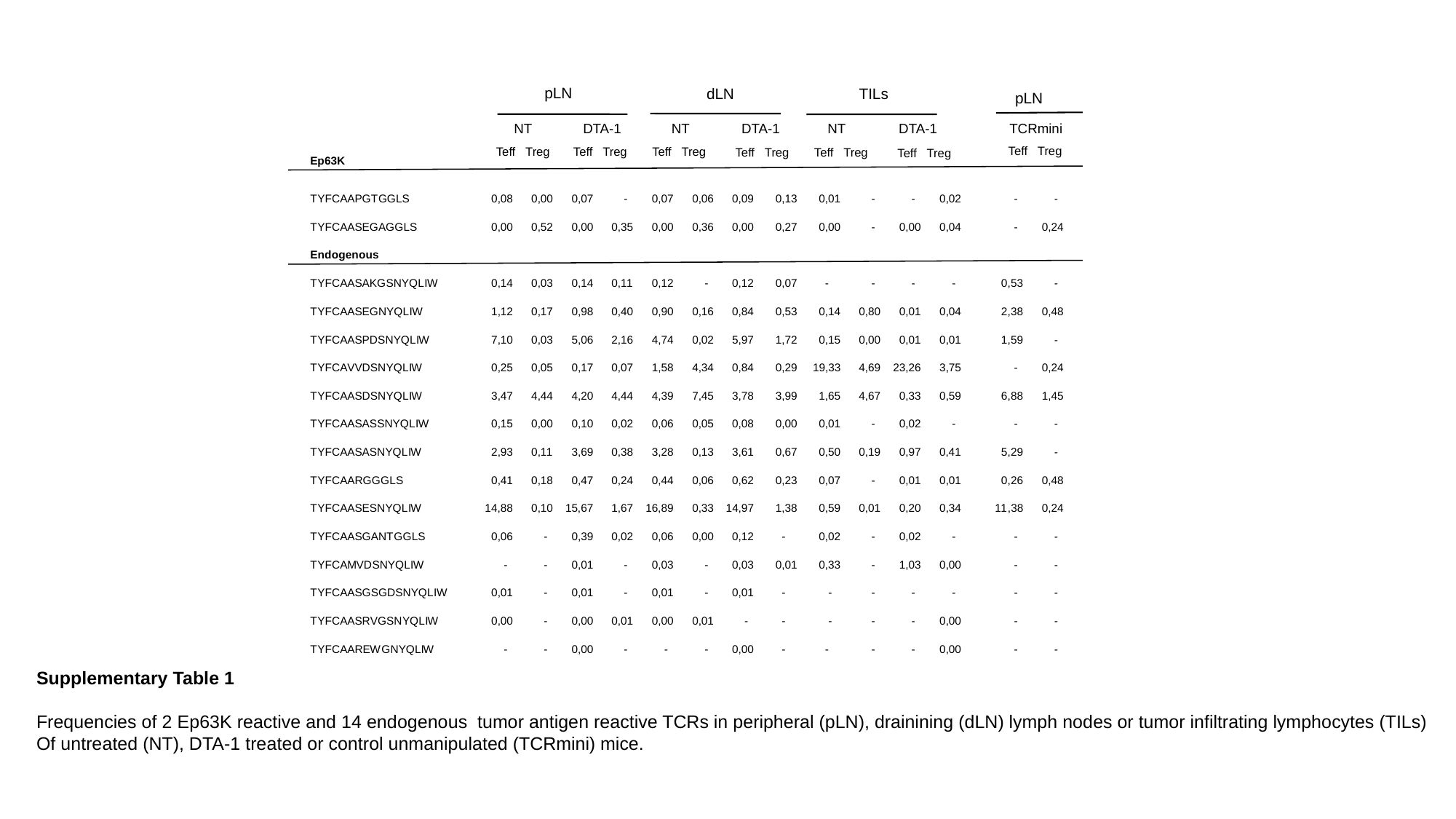

pLN
dLN
TILs
pLN
DTA-1
TCRmini
NT
DTA-1
NT
DTA-1
NT
Teff Treg
Teff Treg
Teff Treg
Teff Treg
Teff Treg
Teff Treg
Teff Treg
Supplementary Table 1
Frequencies of 2 Ep63K reactive and 14 endogenous tumor antigen reactive TCRs in peripheral (pLN), drainining (dLN) lymph nodes or tumor infiltrating lymphocytes (TILs)
Of untreated (NT), DTA-1 treated or control unmanipulated (TCRmini) mice.
