# Supplementary material for: Anti-GITR Antibody Treatment Increases TCR Repertoire Diversity of Regulatory but not Effector T Cells Engaged in the Immune Response Against B16 Melanoma
Source: Arch Immunol Ther Exp (Warsz). 2017 Jun 21;65(6):553–64. doi: 10.1007/s00005-017-0479-1 (PMC5688217; doi:10.1007/s00005-017-0479-1)
Supplement: Supplementary file 4 — Supplementary material 4 (PPT 1222 kb) [file 5_2017_479_MOESM4_ESM.ppt]

## Slide 1
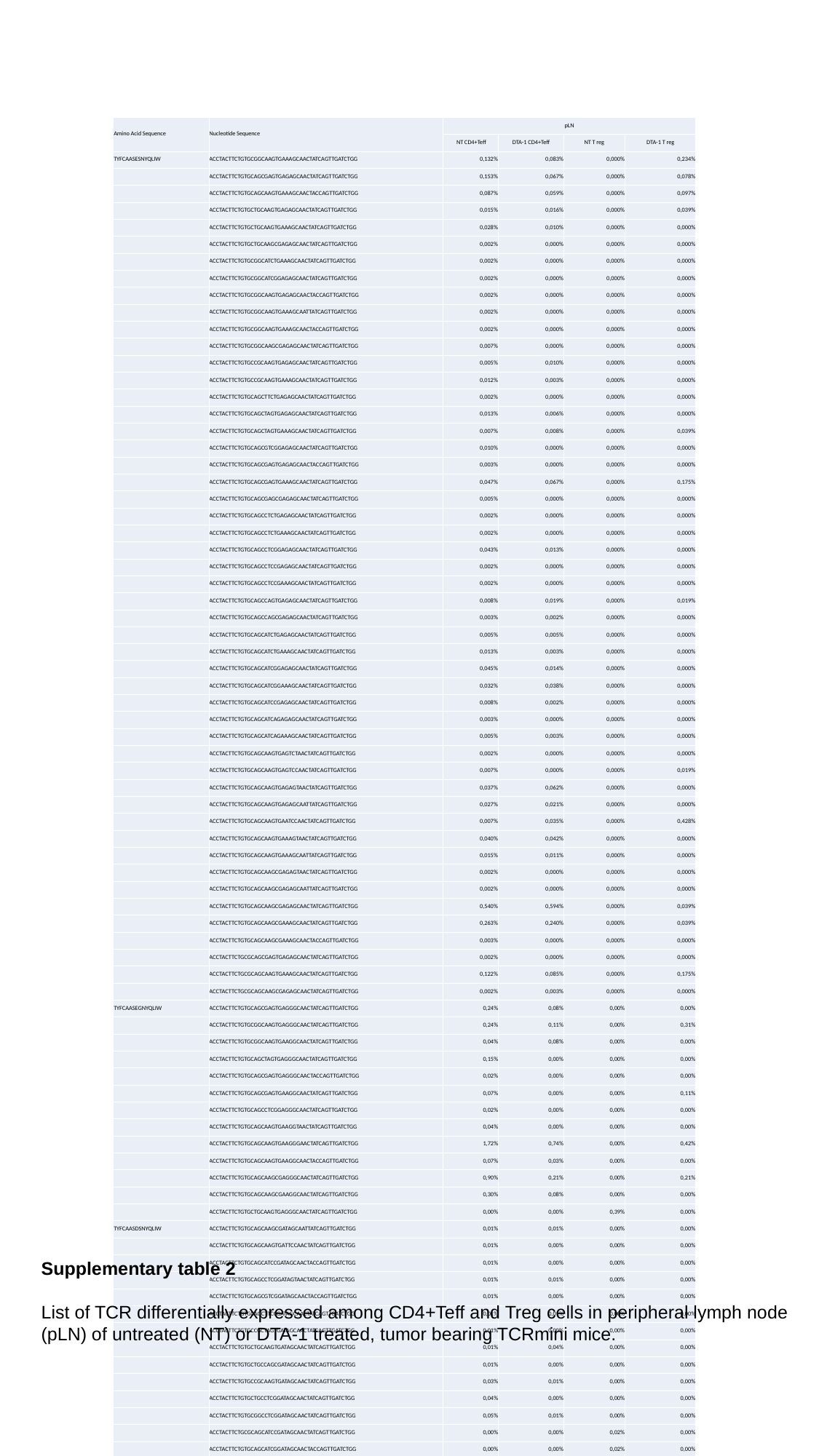

| Amino Acid Sequence | Nucleotide Sequence | pLN | | | |
| --- | --- | --- | --- | --- | --- |
| | | NT CD4+Teff | DTA-1 CD4+Teff | NT T reg | DTA-1 T reg |
| TYFCAASESNYQLIW | ACCTACTTCTGTGCGGCAAGTGAAAGCAACTATCAGTTGATCTGG | 0,132% | 0,083% | 0,000% | 0,234% |
| | ACCTACTTCTGTGCAGCGAGTGAGAGCAACTATCAGTTGATCTGG | 0,153% | 0,067% | 0,000% | 0,078% |
| | ACCTACTTCTGTGCAGCAAGTGAAAGCAACTACCAGTTGATCTGG | 0,087% | 0,059% | 0,000% | 0,097% |
| | ACCTACTTCTGTGCTGCAAGTGAGAGCAACTATCAGTTGATCTGG | 0,015% | 0,016% | 0,000% | 0,039% |
| | ACCTACTTCTGTGCTGCAAGTGAAAGCAACTATCAGTTGATCTGG | 0,028% | 0,010% | 0,000% | 0,000% |
| | ACCTACTTCTGTGCTGCAAGCGAGAGCAACTATCAGTTGATCTGG | 0,002% | 0,000% | 0,000% | 0,000% |
| | ACCTACTTCTGTGCGGCATCTGAAAGCAACTATCAGTTGATCTGG | 0,002% | 0,000% | 0,000% | 0,000% |
| | ACCTACTTCTGTGCGGCATCGGAGAGCAACTATCAGTTGATCTGG | 0,002% | 0,000% | 0,000% | 0,000% |
| | ACCTACTTCTGTGCGGCAAGTGAGAGCAACTACCAGTTGATCTGG | 0,002% | 0,000% | 0,000% | 0,000% |
| | ACCTACTTCTGTGCGGCAAGTGAAAGCAATTATCAGTTGATCTGG | 0,002% | 0,000% | 0,000% | 0,000% |
| | ACCTACTTCTGTGCGGCAAGTGAAAGCAACTACCAGTTGATCTGG | 0,002% | 0,000% | 0,000% | 0,000% |
| | ACCTACTTCTGTGCGGCAAGCGAGAGCAACTATCAGTTGATCTGG | 0,007% | 0,000% | 0,000% | 0,000% |
| | ACCTACTTCTGTGCCGCAAGTGAGAGCAACTATCAGTTGATCTGG | 0,005% | 0,010% | 0,000% | 0,000% |
| | ACCTACTTCTGTGCCGCAAGTGAAAGCAACTATCAGTTGATCTGG | 0,012% | 0,003% | 0,000% | 0,000% |
| | ACCTACTTCTGTGCAGCTTCTGAGAGCAACTATCAGTTGATCTGG | 0,002% | 0,000% | 0,000% | 0,000% |
| | ACCTACTTCTGTGCAGCTAGTGAGAGCAACTATCAGTTGATCTGG | 0,013% | 0,006% | 0,000% | 0,000% |
| | ACCTACTTCTGTGCAGCTAGTGAAAGCAACTATCAGTTGATCTGG | 0,007% | 0,008% | 0,000% | 0,039% |
| | ACCTACTTCTGTGCAGCGTCGGAGAGCAACTATCAGTTGATCTGG | 0,010% | 0,000% | 0,000% | 0,000% |
| | ACCTACTTCTGTGCAGCGAGTGAGAGCAACTACCAGTTGATCTGG | 0,003% | 0,000% | 0,000% | 0,000% |
| | ACCTACTTCTGTGCAGCGAGTGAAAGCAACTATCAGTTGATCTGG | 0,047% | 0,067% | 0,000% | 0,175% |
| | ACCTACTTCTGTGCAGCGAGCGAGAGCAACTATCAGTTGATCTGG | 0,005% | 0,000% | 0,000% | 0,000% |
| | ACCTACTTCTGTGCAGCCTCTGAGAGCAACTATCAGTTGATCTGG | 0,002% | 0,000% | 0,000% | 0,000% |
| | ACCTACTTCTGTGCAGCCTCTGAAAGCAACTATCAGTTGATCTGG | 0,002% | 0,000% | 0,000% | 0,000% |
| | ACCTACTTCTGTGCAGCCTCGGAGAGCAACTATCAGTTGATCTGG | 0,043% | 0,013% | 0,000% | 0,000% |
| | ACCTACTTCTGTGCAGCCTCCGAGAGCAACTATCAGTTGATCTGG | 0,002% | 0,000% | 0,000% | 0,000% |
| | ACCTACTTCTGTGCAGCCTCCGAAAGCAACTATCAGTTGATCTGG | 0,002% | 0,000% | 0,000% | 0,000% |
| | ACCTACTTCTGTGCAGCCAGTGAGAGCAACTATCAGTTGATCTGG | 0,008% | 0,019% | 0,000% | 0,019% |
| | ACCTACTTCTGTGCAGCCAGCGAGAGCAACTATCAGTTGATCTGG | 0,003% | 0,002% | 0,000% | 0,000% |
| | ACCTACTTCTGTGCAGCATCTGAGAGCAACTATCAGTTGATCTGG | 0,005% | 0,005% | 0,000% | 0,000% |
| | ACCTACTTCTGTGCAGCATCTGAAAGCAACTATCAGTTGATCTGG | 0,013% | 0,003% | 0,000% | 0,000% |
| | ACCTACTTCTGTGCAGCATCGGAGAGCAACTATCAGTTGATCTGG | 0,045% | 0,014% | 0,000% | 0,000% |
| | ACCTACTTCTGTGCAGCATCGGAAAGCAACTATCAGTTGATCTGG | 0,032% | 0,038% | 0,000% | 0,000% |
| | ACCTACTTCTGTGCAGCATCCGAGAGCAACTATCAGTTGATCTGG | 0,008% | 0,002% | 0,000% | 0,000% |
| | ACCTACTTCTGTGCAGCATCAGAGAGCAACTATCAGTTGATCTGG | 0,003% | 0,000% | 0,000% | 0,000% |
| | ACCTACTTCTGTGCAGCATCAGAAAGCAACTATCAGTTGATCTGG | 0,005% | 0,003% | 0,000% | 0,000% |
| | ACCTACTTCTGTGCAGCAAGTGAGTCTAACTATCAGTTGATCTGG | 0,002% | 0,000% | 0,000% | 0,000% |
| | ACCTACTTCTGTGCAGCAAGTGAGTCCAACTATCAGTTGATCTGG | 0,007% | 0,000% | 0,000% | 0,019% |
| | ACCTACTTCTGTGCAGCAAGTGAGAGTAACTATCAGTTGATCTGG | 0,037% | 0,062% | 0,000% | 0,000% |
| | ACCTACTTCTGTGCAGCAAGTGAGAGCAATTATCAGTTGATCTGG | 0,027% | 0,021% | 0,000% | 0,000% |
| | ACCTACTTCTGTGCAGCAAGTGAATCCAACTATCAGTTGATCTGG | 0,007% | 0,035% | 0,000% | 0,428% |
| | ACCTACTTCTGTGCAGCAAGTGAAAGTAACTATCAGTTGATCTGG | 0,040% | 0,042% | 0,000% | 0,000% |
| | ACCTACTTCTGTGCAGCAAGTGAAAGCAATTATCAGTTGATCTGG | 0,015% | 0,011% | 0,000% | 0,000% |
| | ACCTACTTCTGTGCAGCAAGCGAGAGTAACTATCAGTTGATCTGG | 0,002% | 0,000% | 0,000% | 0,000% |
| | ACCTACTTCTGTGCAGCAAGCGAGAGCAATTATCAGTTGATCTGG | 0,002% | 0,000% | 0,000% | 0,000% |
| | ACCTACTTCTGTGCAGCAAGCGAGAGCAACTATCAGTTGATCTGG | 0,540% | 0,594% | 0,000% | 0,039% |
| | ACCTACTTCTGTGCAGCAAGCGAAAGCAACTATCAGTTGATCTGG | 0,263% | 0,240% | 0,000% | 0,039% |
| | ACCTACTTCTGTGCAGCAAGCGAAAGCAACTACCAGTTGATCTGG | 0,003% | 0,000% | 0,000% | 0,000% |
| | ACCTACTTCTGCGCAGCGAGTGAGAGCAACTATCAGTTGATCTGG | 0,002% | 0,000% | 0,000% | 0,000% |
| | ACCTACTTCTGCGCAGCAAGTGAAAGCAACTATCAGTTGATCTGG | 0,122% | 0,085% | 0,000% | 0,175% |
| | ACCTACTTCTGCGCAGCAAGCGAGAGCAACTATCAGTTGATCTGG | 0,002% | 0,003% | 0,000% | 0,000% |
| TYFCAASEGNYQLIW | ACCTACTTCTGTGCAGCGAGTGAGGGCAACTATCAGTTGATCTGG | 0,24% | 0,08% | 0,00% | 0,00% |
| | ACCTACTTCTGTGCGGCAAGTGAGGGCAACTATCAGTTGATCTGG | 0,24% | 0,11% | 0,00% | 0,31% |
| | ACCTACTTCTGTGCGGCAAGTGAAGGCAACTATCAGTTGATCTGG | 0,04% | 0,08% | 0,00% | 0,00% |
| | ACCTACTTCTGTGCAGCTAGTGAGGGCAACTATCAGTTGATCTGG | 0,15% | 0,00% | 0,00% | 0,00% |
| | ACCTACTTCTGTGCAGCGAGTGAGGGCAACTACCAGTTGATCTGG | 0,02% | 0,00% | 0,00% | 0,00% |
| | ACCTACTTCTGTGCAGCGAGTGAAGGCAACTATCAGTTGATCTGG | 0,07% | 0,00% | 0,00% | 0,11% |
| | ACCTACTTCTGTGCAGCCTCGGAGGGCAACTATCAGTTGATCTGG | 0,02% | 0,00% | 0,00% | 0,00% |
| | ACCTACTTCTGTGCAGCAAGTGAAGGTAACTATCAGTTGATCTGG | 0,04% | 0,00% | 0,00% | 0,00% |
| | ACCTACTTCTGTGCAGCAAGTGAAGGGAACTATCAGTTGATCTGG | 1,72% | 0,74% | 0,00% | 0,42% |
| | ACCTACTTCTGTGCAGCAAGTGAAGGCAACTACCAGTTGATCTGG | 0,07% | 0,03% | 0,00% | 0,00% |
| | ACCTACTTCTGTGCAGCAAGCGAGGGCAACTATCAGTTGATCTGG | 0,90% | 0,21% | 0,00% | 0,21% |
| | ACCTACTTCTGTGCAGCAAGCGAAGGCAACTATCAGTTGATCTGG | 0,30% | 0,08% | 0,00% | 0,00% |
| | ACCTACTTCTGTGCTGCAAGTGAGGGCAACTATCAGTTGATCTGG | 0,00% | 0,00% | 0,39% | 0,00% |
| TYFCAASDSNYQLIW | ACCTACTTCTGTGCAGCAAGCGATAGCAATTATCAGTTGATCTGG | 0,01% | 0,01% | 0,00% | 0,00% |
| | ACCTACTTCTGTGCAGCAAGTGATTCCAACTATCAGTTGATCTGG | 0,01% | 0,00% | 0,00% | 0,00% |
| | ACCTACTTCTGTGCAGCATCCGATAGCAACTACCAGTTGATCTGG | 0,01% | 0,00% | 0,00% | 0,00% |
| | ACCTACTTCTGTGCAGCCTCGGATAGTAACTATCAGTTGATCTGG | 0,01% | 0,01% | 0,00% | 0,00% |
| | ACCTACTTCTGTGCAGCGTCGGATAGCAACTACCAGTTGATCTGG | 0,01% | 0,00% | 0,00% | 0,00% |
| | ACCTACTTCTGTGCAGCTTCCGATAGCAACTATCAGTTGATCTGG | 0,01% | 0,01% | 0,00% | 0,00% |
| | ACCTACTTCTGTGCCGCTAGTGATAGCAACTATCAGTTGATCTGG | 0,01% | 0,00% | 0,00% | 0,00% |
| | ACCTACTTCTGTGCTGCAAGTGATAGCAACTATCAGTTGATCTGG | 0,01% | 0,04% | 0,00% | 0,00% |
| | ACCTACTTCTGTGCTGCCAGCGATAGCAACTATCAGTTGATCTGG | 0,01% | 0,00% | 0,00% | 0,00% |
| | ACCTACTTCTGTGCCGCAAGTGATAGCAACTATCAGTTGATCTGG | 0,03% | 0,01% | 0,00% | 0,00% |
| | ACCTACTTCTGTGCTGCCTCGGATAGCAACTATCAGTTGATCTGG | 0,04% | 0,00% | 0,00% | 0,00% |
| | ACCTACTTCTGTGCGGCCTCGGATAGCAACTATCAGTTGATCTGG | 0,05% | 0,01% | 0,00% | 0,00% |
| | ACCTACTTCTGCGCAGCATCCGATAGCAACTATCAGTTGATCTGG | 0,00% | 0,00% | 0,02% | 0,00% |
| | ACCTACTTCTGTGCAGCATCGGATAGCAACTACCAGTTGATCTGG | 0,00% | 0,00% | 0,02% | 0,00% |
| | ACCTACTTCTGTGCGGCATCCGATAGCAACTATCAGTTGATCTGG | 0,00% | 0,00% | 0,04% | 0,00% |
| TYFCAASASNYQLIW | ACCTACTTCTGCGCGGCAAGTGCTAGCAACTATCAGTTGATCTGG | 0,02% | 0,00% | 0,00% | 0,00% |
| | ACCTACTTCTGTGCAGCAAGCGCAAGCAACTATCAGTTGATCTGG | 0,05% | 0,10% | 0,00% | 0,00% |
| | ACCTACTTCTGTGCAGCAAGCGCCTCCAACTATCAGTTGATCTGG | 0,03% | 0,00% | 0,00% | 0,00% |
| | ACCTACTTCTGTGCAGCAAGCGCTAGCAACTACCAGTTGATCTGG | 0,02% | 0,00% | 0,00% | 0,00% |
| | ACCTACTTCTGTGCAGCAAGCGCTAGCAACTATCAGTTGATCTGG | 0,25% | 0,24% | 0,00% | 0,00% |
| | ACCTACTTCTGTGCAGCAAGTGCATCCAACTATCAGTTGATCTGG | 0,05% | 0,02% | 0,00% | 0,00% |
| | ACCTACTTCTGTGCAGCAAGTGCCAGCAACTACCAGTTGATCTGG | 0,01% | 0,01% | 0,00% | 0,00% |
| | ACCTACTTCTGTGCAGCAAGTGCCAGCAATTATCAGTTGATCTGG | 0,01% | 0,02% | 0,00% | 0,00% |
| | ACCTACTTCTGTGCAGCAAGTGCGAGTAACTATCAGTTGATCTGG | 0,03% | 0,00% | 0,00% | 0,00% |
| | ACCTACTTCTGTGCAGCAAGTGCTAGTAACTATCAGTTGATCTGG | 0,04% | 0,01% | 0,00% | 0,00% |
| | ACCTACTTCTGTGCAGCATCCGCTAGCAACTATCAGTTGATCTGG | 0,01% | 0,00% | 0,00% | 0,00% |
| | ACCTACTTCTGTGCAGCATCTGCTAGCAACTATCAGTTGATCTGG | 0,01% | 0,00% | 0,00% | 0,00% |
| | ACCTACTTCTGTGCAGCCTCGGCTAGCAACTATCAGTTGATCTGG | 0,01% | 0,00% | 0,00% | 0,00% |
| | ACCTACTTCTGTGCAGCGAGCGCTAGCAACTATCAGTTGATCTGG | 0,01% | 0,00% | 0,00% | 0,00% |
| | ACCTACTTCTGTGCAGCGAGTGCCAGCAACTATCAGTTGATCTGG | 0,03% | 0,01% | 0,00% | 0,00% |
| | ACCTACTTCTGTGCAGCGAGTGCTAGCAACTATCAGTTGATCTGG | 0,18% | 0,03% | 0,00% | 0,00% |
| | ACCTACTTCTGTGCAGCGTCGGCTAGCAACTATCAGTTGATCTGG | 0,02% | 0,00% | 0,00% | 0,00% |
| | ACCTACTTCTGTGCAGCTAGTGCCAGCAACTATCAGTTGATCTGG | 0,01% | 0,00% | 0,00% | 0,00% |
| | ACCTACTTCTGTGCCGCAAGTGCGAGCAACTATCAGTTGATCTGG | 0,02% | 0,00% | 0,00% | 0,00% |
| | ACCTACTTCTGTGCGGCAAGTGCCAGCAACTATCAGTTGATCTGG | 0,04% | 0,04% | 0,00% | 0,00% |
| | ACCTACTTCTGTGCGGCAAGTGCTAGCAACTATCAGTTGATCTGG | 0,09% | 0,02% | 0,00% | 0,00% |
| | ACCTACTTCTGTGCTGCAAGTGCAAGCAACTATCAGTTGATCTGG | 0,01% | 0,01% | 0,00% | 0,00% |
| TYFCAMVDSNYQLIW | ACCTACTTCTGTGCAATGGTGGATAGCAACTATCAGTTGATCTGG | 100,00% | 97,44% | 0,00% | 0,00% |
| TYFCAVVDSNYQLIW | ACCTACTTCTGTGCAGTGGTGGATAGCAACTATCAGTTGATCTGG | 35,60% | 33,96% | 0,00% | 34,11% |
| | ACCTACTTCTGTGCAGTAGTGGATAGCAACTATCAGTTGATCTGG | 10,20% | 8,66% | 0,00% | 0,00% |
| | ACCTACTTCTGTGCGGTTGTGGATAGCAACTATCAGTTGATCTGG | 1,51% | 1,20% | 0,00% | 0,00% |
| | ACCTACTTCTGTGCAGTCGTAGATAGCAACTATCAGTTGATCTGG | 1,32% | 0,00% | 0,00% | 24,77% |
| | ACCTACTTCTGTGCTGTGGTGGATAGCAACTATCAGTTGATCTGG | 1,04% | 3,06% | 0,00% | 0,00% |
| | ACCTACTTCTGTGCAGTAGTAGATAGCAACTATCAGTTGATCTGG | 1,04% | 0,00% | 0,00% | 0,00% |
| | ACCTACTTCTGTGCGGTTGTCGATAGCAACTATCAGTTGATCTGG | 0,76% | 0,00% | 0,00% | 0,00% |
| | ACCTACTTCTGTGCGGTCGTGGATAGCAACTATCAGTTGATCTGG | 0,66% | 0,27% | 0,00% | 0,00% |
| | ACCTACTTCTGTGCAGTAGTCGATAGCAACTATCAGTTGATCTGG | 0,66% | 1,47% | 0,00% | 0,00% |
| | ACCTACTTCTGTGCAGTGGTCGATAGCAACTATCAGTTGATCTGG | 0,47% | 0,13% | 0,00% | 0,00% |
| | ACCTACTTCTGTGCCGTTGTGGATAGCAACTATCAGTTGATCTGG | 0,38% | 0,67% | 0,00% | 0,00% |
| | ACCTACTTCTGTGCAGTGGTAGATAGCAACTATCAGTTGATCTGG | 0,38% | 0,00% | 0,00% | 0,00% |
| | ACCTACTTCTGTGCTGTTGTGGATAGCAACTATCAGTTGATCTGG | 0,28% | 0,00% | 0,00% | 0,00% |
| | ACCTACTTCTGTGCGGTAGTGGATAGCAACTATCAGTTGATCTGG | 0,28% | 0,80% | 0,00% | 0,00% |
| | ACCTACTTCTGTGCAGTGGTTGATAGCAACTATCAGTTGATCTGG | 0,19% | 0,00% | 0,00% | 0,00% |
| | ACCTACTTCTGTGCAGTGGTGGACAGCAACTATCAGTTGATCTGG | 0,19% | 0,00% | 0,00% | 0,00% |
| | ACCTACTTCTGTGCCGTCGTGGATAGCAACTATCAGTTGATCTGG | 0,09% | 0,93% | 0,00% | 0,00% |
| | ACCTACTTCTGTGCAGTCGTCGACAGCAACTATCAGTTGATCTGG | 0,09% | 0,00% | 0,00% | 0,00% |
| | ACCTACTTCTGTGCGGTCGTTGATAGCAACTATCAGTTGATCTGG | 0,09% | 0,00% | 0,00% | 0,00% |
| | ACCTACTTCTGTGCCGTGGTGGATAGTAACTATCAGTTGATCTGG | 0,09% | 0,00% | 0,00% | 0,00% |
| | ACCTACTTCTGCGCAGTCGTTGATAGCAACTATCAGTTGATCTGG | 0,09% | 0,00% | 0,00% | 0,00% |
| | ACCTACTTCTGTGCAGTCGTGGATAGCAACTACCAGTTGATCTGG | 0,00% | 0,00% | 1,27% | 0,00% |
| | | | | | |
Supplementary table 2
List of TCR differentially expressed among CD4+Teff and Treg cells in peripheral lymph node
(pLN) of untreated (NT) or DTA-1 treated, tumor bearing TCRmini mice.
